# Functional personalization in digital manufacturing: a translational framework for patient-specific implants, arthroscopic locators, and therapeutic constructs

**DOI:** 10.3389/fbioe.2026.1903071

**Published:** 2026-09-28

**Authors:** Qun Li, Yi-Han Yang, Jin-Wei Chu, Xu-Yang Wang, Song-Ying Du, Hong-Gang Zhu, Huai-Yu Yang

**Affiliations:** 1 Department of Orthopaedic Surgery, General Hospital of Fuxin Coal Mine, Liaoning Health Industry Group (Department of Joint and Sports Medicine), Fuxin, Liaoning, China; 2 Design Program, School of Arts, Guilin University of Technology, Guilin, China

**Keywords:** 3D printing, anterior cruciate ligament reconstruction, arthroscopic locator, artificial intelligence, digital manufacturing, digital twin, knee arthroscopy, patient-specific implant

## Abstract

Three-dimensional printing and digital manufacturing are increasingly used to produce patient-specific implants, surgical guides, arthroscopic locators, and therapeutic constructs. Yet the field is often described through a device-centered lens, emphasizing anatomical customization, printing accuracy, and material feasibility. This framing is becoming insufficient. Patient-specific manufacturing should not be understood merely as the fabrication of individualized objects, but as a clinically executable workflow that translates imaging data into surgical action, regenerative therapy, and outcome-informed iteration. This Perspective proposes that the next stage of 3D printing in medicine depends on three conceptual shifts: from anatomical matching to functional and procedural optimization; from passive printed products to interfaces that carry digital planning into the operating room; and from isolated fabrication success to clinically validated, regulation-aware, and feedback-driven manufacturing systems. Orthopedic applications are used as a representative context, with particular attention to 3D-printed arthroscopic locators for knee arthroscopic anterior cruciate ligament reconstruction. This example illustrates how patient-specific design can move beyond open reconstruction into minimally invasive procedures, where accurate localization, reproducibility, and workflow compatibility are central. We further discuss therapeutic constructs, artificial intelligence, digital twins, point-of-care manufacturing, and translational barriers. Rather than offering another broad review of 3D printing applications, this Perspective proposes a framework for defining success in patient-specific digital manufacturing as the reliable conversion of patient data into safe, reproducible, and clinically meaningful intervention.

## 1 Introduction

Three-dimensional printing has become one of the most visible technologies in personalized medicine (Serrano et al., 2023). In orthopedics, craniofacial surgery, spinal reconstruction, tissue engineering, and surgical planning, additive manufacturing has enabled the production of anatomical models, customized implants, surgical guides, porous scaffolds, and biofabricated constructs (Anderson et al., 2022; Mancilla-De-la-Cruz et al., 2022; Chen T. et al., 2025). These developments have been widely interpreted as evidence that medicine is moving from standardized devices toward patient-specific solutions. This interpretation is correct, but incomplete. The deeper significance of 3D printing is not only that a device can be made to match an individual patient’s anatomy, but that a digital clinical plan can be converted into a physical tool or construct that changes how surgery and therapy are executed (Sousa et al., 2025).

This distinction matters because much of the current discussion around medical 3D printing remains product-centered. A patient-specific implant is often evaluated by whether it fits the defect (Alshehri et al., 2021). A surgical guide is judged by whether it reproduces a planned trajectory (Huang et al., 2020). A scaffold is assessed by whether it can be printed with appropriate pores, shape, or material composition (Valtonen et al., 2020). These are necessary considerations, but they do not capture the full translational challenge. In clinical practice, a printed device must be embedded within a larger chain of decisions: image acquisition, segmentation, virtual planning, computer-aided design, simulation, fabrication, post-processing, sterilization, intraoperative use, postoperative evaluation, and long-term outcome assessment. A failure at any point in this chain can limit the value of the final product (Zhang, 2023; Li et al., 2025; Couți et al., 2025).

We therefore believe that patient-specific digital manufacturing—defined here as an integrated workflow that uses patient data, digital planning, computer-aided design, and additive or digital fabrication to create individualized clinical tools, devices, or therapeutic constructs—should now be reframed. The central question is no longer simply whether individualized implants, surgical guides, or therapeutic constructs can be printed. The more important question is whether these technologies can create clinically executable interventions that are accurate, reproducible, biologically meaningful, regulation-compatible, and scalable across health systems (Mei et al., 2022; Kim et al., 2025). This reframing is particularly relevant to the Research Topic “Health System Innovation Enabled by Artificial Intelligence, Digital Health, and Advanced Materials (Zhao et al., 2026)” because 3D printing sits at the intersection of digital health, computational planning, advanced biomaterials, and clinical implementation (ACS, 2020).

For clarity, four key terms are used consistently throughout this Perspective. An “arthroscopic locator” refers to a compact, patient-specific physical guide designed to register with predefined anatomical features and transfer a planned position or trajectory into a constrained arthroscopic field. A “therapeutic construct” refers to a digitally manufactured scaffold, implant, or biofabricated system that combines spatial architecture with materials, drugs, cells, or bioactive signals to actively support or regulate tissue repair rather than providing structural replacement alone. A “digital twin” refers to a patient-specific computational representation of anatomy, mechanics, physiology, or an intervention that can simulate clinically relevant behavior and be updated as new intraoperative or postoperative data become available. “Function matching” refers to tailoring a patient-specific product to the mechanical, biological, and procedural requirements of its intended clinical task, rather than limiting customization to geometric conformity.

Orthopedics provides a useful lens through which to examine this transition. Bone and joint diseases often involve complex anatomy, high mechanical demands, and procedure-dependent outcomes (McCormack et al., 2018). 3D-printed implants can restore irregular defects (Luis et al., 2020); porous scaffolds can support bone ingrowth (Deng et al., 2021); patient-specific surgical guides can improve osteotomy or screw placement (Borbon et al., 2025); and digital manufacturing can generate constructs for cartilage, osteochondral, or bone regeneration (Yang et al., 2025). More recently, 3D printing has entered minimally invasive orthopedic surgery. In knee arthroscopy, patient-specific arthroscopic locators have been developed to assist femoral tunnel positioning during anterior cruciate ligament reconstruction (Qu et al., 2024). This example is especially important because it shows that patient-specific manufacturing is not limited to large open implants. It can also act as a bridge between preoperative digital planning and intraoperative decision-making in constrained arthroscopic spaces.

The purpose of this Perspective is not to provide another comprehensive review of 3D printing technologies. Instead, we focus on a conceptual and translational gap: how should success in patient-specific digital manufacturing be defined? Success should not be measured only by whether a device is printable, anatomically matched, or mechanically feasible. Rather, it should be measured by whether digital manufacturing can reliably convert patient data into safe, reproducible, and outcome-improving interventions (Choi et al., 2026). To support this argument, we discuss three related domains: patient-specific implants, surgical guides and arthroscopic locators, and therapeutic constructs. We then consider how artificial intelligence, digital twins, and point-of-care manufacturing may reshape the field, while also emphasizing the regulatory, quality-control, and evidence-generation barriers that must be addressed.

This structure follows a deliberate progression from structure, to procedure, to biology. Patient-specific implants illustrate how digital manufacturing can restore or optimize anatomical and mechanical structures. Surgical locators then show how printed tools can transfer a digital plan into procedural action, particularly in constrained operative environments. Therapeutic constructs further extend this logic by using printed architecture, materials, cells, or bioactive signals to guide biological repair. This progression provides the organizing logic for the following sections and reflects the broader movement of the field from customized objects toward clinically executable interventions.

### 1.1 Positioning and novelty of this perspective

Recent reviews have summarized medical 3D printing, additive manufacturing of implants, surgical guides, bioprinting, and tissue engineering scaffolds (Muhindo et al., 2023; Hariharan et al., 2025). These reviews are valuable because they map the technological landscape and describe the diversity of available methods. However, many are organized around materials, printers, anatomical regions, or device categories (Zhang, 2022). Such organization can unintentionally reinforce the view that the field is primarily about producing customized objects.

The present Perspective takes a different position. We propose that patient-specific digital manufacturing should be understood as a translational workflow and clinical execution system. In this view, the printed implant, surgical guide, arthroscopic locator, or therapeutic construct is only one part of a broader digital-to-physical continuum. What matters is not simply whether a device reproduces anatomy, but whether it carries a validated plan into surgery or therapy, performs reliably under real clinical constraints, and generates outcome data that can improve future designs (Zhang et al., 2023).

The novelty of this Perspective therefore lies in integrating three dimensions that are often discussed separately: device personalization, procedural execution, and translational governance. By linking these dimensions, we propose a framework that complements technology-centered reviews and helps reorient the field from “what can be printed?” toward “what should be clinically executed, validated, and improved?”

Representative applications of patient-specific digital manufacturing, including implants, surgical guides, arthroscopic locators, and therapeutic constructs, are summarized in Supplementary Table S1 to clarify how different printed products contribute to integrated clinical workflows rather than isolated device fabrication.

## 2 Patient-specific manufacturing should move beyond anatomical matching

### 2.1 Anatomical fit is necessary, but no longer sufficient

The first major clinical advantage of 3D printing is the ability to produce devices that match patient anatomy. This is especially valuable in settings where standardized devices cannot adequately address irregular defects, complex deformities, or individualized skeletal contours. In craniofacial reconstruction, pelvic surgery, spinal surgery, orthopedic oncology, and revision procedures, patient-specific implants can reduce the need for intraoperative reshaping and may improve contact between the implant and host tissue (Döring et al., 2021; Kalmar et al., 2020; Laubach et al., 2025). This capacity remains foundational to the field.

However, anatomical fit should now be regarded as the starting point rather than the endpoint of digital manufacturing. A device that fits the patient’s geometry may still fail if it does not meet mechanical, biological, surgical, or regulatory requirements. For example, a bone implant must not only match the defect but also distribute load appropriately, avoid excessive stiffness mismatch, support osseointegration, tolerate sterilization, and maintain long-term integrity (Zhang et al., 2021). Similarly, a scaffold for cartilage or osteochondral repair must not only fill a defect but also support cell behavior, matrix formation, nutrient transport, and tissue-specific mechanical function (Liu et al., 2026).

This distinction becomes particularly important as the field moves toward more advanced implants and therapeutic constructs. Additive manufacturing allows control over pore architecture, lattice design, surface roughness, regional stiffness, and material distribution (Ma et al., 2025). These parameters make it possible to design implants that are not merely patient-shaped but patient-optimized. In principle, a construct can be tailored to a patient’s anatomy, mechanical environment, tissue quality, surgical plan, and biological risk (Garot et al., 2020). This represents a conceptual shift from “shape matching” to “function matching.”

Function matching should also be evaluated through task-specific and measurable endpoints. For load-bearing implants, relevant metrics may include implant–bone contact area, stiffness mismatch, stress distribution, fatigue resistance, interfacial micromotion, radiographic osseointegration, implant loosening, revision rate, pain scores, and functional recovery (Laubach et al., 2025; Zhang et al., 2021). For surgical locators, objective assessment may include deviation between planned and achieved entry points or trajectories, registration error, docking stability, number of positioning attempts, operative time, intraoperative usability, and procedure-related complications (Zhang et al., 2025; Gong and Yin, 2025). For scaffolds or therapeutic constructs, functional endpoints may include pore interconnectivity, compressive modulus, degradation rate, cell viability, matrix deposition, vascularization, defect filling on imaging, histological repair quality, integration with host tissue, and durability of clinical improvement (Liu et al., 2026; Son et al., 2025). These examples show that functional personalization should be judged by whether the individualized design improves mechanical behavior, procedural execution, biological performance, or patient outcomes, rather than by geometric conformity alone.

Future studies should explicitly separate anatomical customization from functional personalization. The former asks whether a device conforms to a patient’s geometry. The latter asks whether the design improves clinical performance under patient-specific conditions. This difference should influence experimental design, outcome measurement, and regulatory evaluation. A patient-specific device should not be considered successful simply because it is individualized; it should be considered successful when its individualization solves a clinically meaningful problem.

### 2.2 Materials should be selected according to clinical tasks, not printing convenience

Material selection remains one of the most important determinants of translational success. Titanium and titanium alloys are widely used for load-bearing orthopedic implants because of their strength, corrosion resistance, and biocompatibility (Li J. et al., 2026). Additive manufacturing further enhances their utility by enabling porous structures that support bone ingrowth and mechanical interlocking (González-Henríquez et al., 2022). PEEK offers a lower elastic modulus closer to cortical bone and has attracted interest for spinal and cranial implants, although its bioinert surface often requires modification (Dondani et al., 2023). Bioceramics such as hydroxyapatite, tricalcium phosphate, and bioactive glass provide osteoconductive properties but are limited by brittleness (Makishi et al., 2023). Biodegradable polymers such as polycaprolactone, polylactic acid, and polylactic-co-glycolic acid are attractive for temporary scaffolds and drug-releasing constructs, but their degradation, mechanics, and inflammatory behavior must be carefully controlled (Samadian et al., 2020).

The choice of material should therefore be governed by the clinical task, not by ease of printing alone. A load-bearing implant, an arthroscopic locator, and a bioactive scaffold have fundamentally different requirements. A permanent implant must satisfy long-term mechanical and biological criteria (Li J. et al., 2026). A temporary surgical guide must prioritize dimensional accuracy, sterilization compatibility, safe tissue contact, and workflow efficiency. A therapeutic construct must additionally address drug release, cell compatibility, degradation kinetics, and local microenvironmental regulation (Joiner et al., 2022). Treating all printed medical products as variations of the same manufacturing problem obscures these differences.

The future of patient-specific manufacturing will increasingly depend on hybrid material strategies. A single material rarely satisfies all clinical needs. A bone repair device may require a load-bearing metallic framework, a porous osteoconductive interface, and an antibacterial or immunomodulatory surface. A cartilage-related construct may require a mechanically supportive polymer framework combined with a hydrogel phase that supports chondrocyte survival and matrix deposition. An arthroscopic locator may require a compact sterilizable polymer with sufficient rigidity, smooth surfaces, and stable docking geometry. Thus, the next stage of material innovation should emphasize task-specific integration rather than material novelty in isolation.

Material selection should therefore be justified by the intended clinical function, duration and type of tissue contact, mechanical environment, and acceptable failure mode. Metallic materials such as titanium or tantalum alloys are appropriate for permanent load-bearing implants because they provide high strength, fatigue resistance, corrosion resistance, and compatibility with porous structures that promote bone ingrowth; their validation should include tensile or compressive properties, fatigue performance, interfacial stability, corrosion and ion-release behavior, and biological responses related to long-term implantation. PEEK may be selected when radiolucency and a modulus closer to cortical bone are desirable, but testing should address creep, fatigue, wear, sterilization stability, surface-dependent osseointegration, and biological safety. Bioceramics are suited to osteoconductive interfaces or defect-filling scaffolds but require assessment of compressive strength, fracture resistance, dissolution behavior, phase stability, and bone-forming responses because of their brittleness. Biodegradable polymers are more appropriate for temporary scaffolds or drug-releasing constructs and should be evaluated for mechanical retention during healing, degradation kinetics, molecular-weight loss, degradation products, local pH changes, residual processing chemicals, inflammatory responses, and tissue integration. For hydrogels, bioinks, and cell-laden constructs, rheology, print fidelity, swelling, degradation, early mechanical stability, cell viability, sterility, endotoxin burden, biological potency, and tissue-specific function become central quality attributes. These tests should be selected through a risk-based biological evaluation rather than applied as an identical checklist to every material or product.

## 3 Surgical guides and arthroscopic locators reveal the procedural value of digital manufacturing

### 3.1 Printed surgical guides are physical interfaces between planning and action

Surgical guides represent one of the clearest examples of digital manufacturing as a clinical execution tool. As defined above, an arthroscopic locator is a compact patient-specific guide intended for localization in constrained arthroscopic spaces, such as ACL femoral tunnel positioning. Here, “surgical guide” is retained as the broader term for printed devices that transfer a planned position, trajectory, or resection margin into surgery, whereas “guide plate” and “surgical template” are not treated as separate device categories. We reserve “arthroscopic locator” for compact patient-specific guides used for localization in constrained arthroscopic spaces, such as ACL femoral tunnel positioning, and do not treat “guide plate” or “surgical template” as separate device categories. Unlike permanent implants, these devices may not remain in the body, but they can strongly influence the accuracy and reproducibility of a procedure. They translate preoperative planning into intraoperative action by guiding cutting, drilling, screw placement, tunnel creation, resection margins, or portal-related localization. Their value lies in making a virtual plan physically usable.

This function is particularly important in procedures where small deviations can have large consequences. In high tibial osteotomy, inaccurate correction may compromise alignment. In spinal surgery, deviation in screw trajectory may threaten neurovascular structures (Zhao et al., 2023). In tumor surgery, imprecise cutting may affect margins or reconstruction fit (Frankel et al., 2025). In ligament reconstruction, tunnel malposition may alter graft behavior and knee kinematics (Zhou et al., 2022). In each case, the surgical guide or locator is not simply a printed accessory; it is an interface that converts digital knowledge into a controlled surgical maneuver.

This idea changes how surgical guides and arthroscopic locators should be evaluated. Accuracy alone is not enough. A clinically meaningful guide or locator must also be easy to position, stable during use, compatible with the surgical exposure, safe for surrounding tissues, sterilizable, and reproducible across cases (Qu et al., 2024). It should reduce rather than increase procedural complexity. These requirements are particularly demanding in minimally invasive surgery, where visibility is limited and working space is constrained.

### 3.2 Knee arthroscopic ACL reconstruction illustrates a new Frontier

Knee arthroscopy provides an important example of how 3D-printed arthroscopic locators can extend patient-specific digital manufacturing into minimally invasive surgery (Glatzeder et al., 2022). Anterior cruciate ligament reconstruction depends heavily on accurate femoral tunnel positioning. Malposition may lead to graft impingement, abnormal graft tension, residual instability, altered knee kinematics, or reconstruction failure (Pintore et al., 2025). Traditional techniques rely on anatomical landmarks, arthroscopic visualization, surgeon experience, and standard surgical guides. Although these methods can produce excellent results, individual anatomical variation and limited arthroscopic visibility can still make tunnel placement challenging.

Recent studies have explored patient-specific 3D-printed femoral tunnel locators for ACL reconstruction. In these workflows, preoperative CT or MRI data are used to reconstruct the distal femur and identify the intended femoral tunnel target (Eljaja et al., 2025). A patient-specific arthroscopic locator is then designed to match the lateral femoral condyle or related anatomical landmarks and to guide the drilling trajectory. During arthroscopy, the locator is introduced through a portal or accessory incision, docked onto the planned region, and used to assist tunnel positioning. Figure 1 provides a detailed illustration of this process, showing how preoperative digital planning is physically transferred into the arthroscopic field.

**FIGURE 1:**
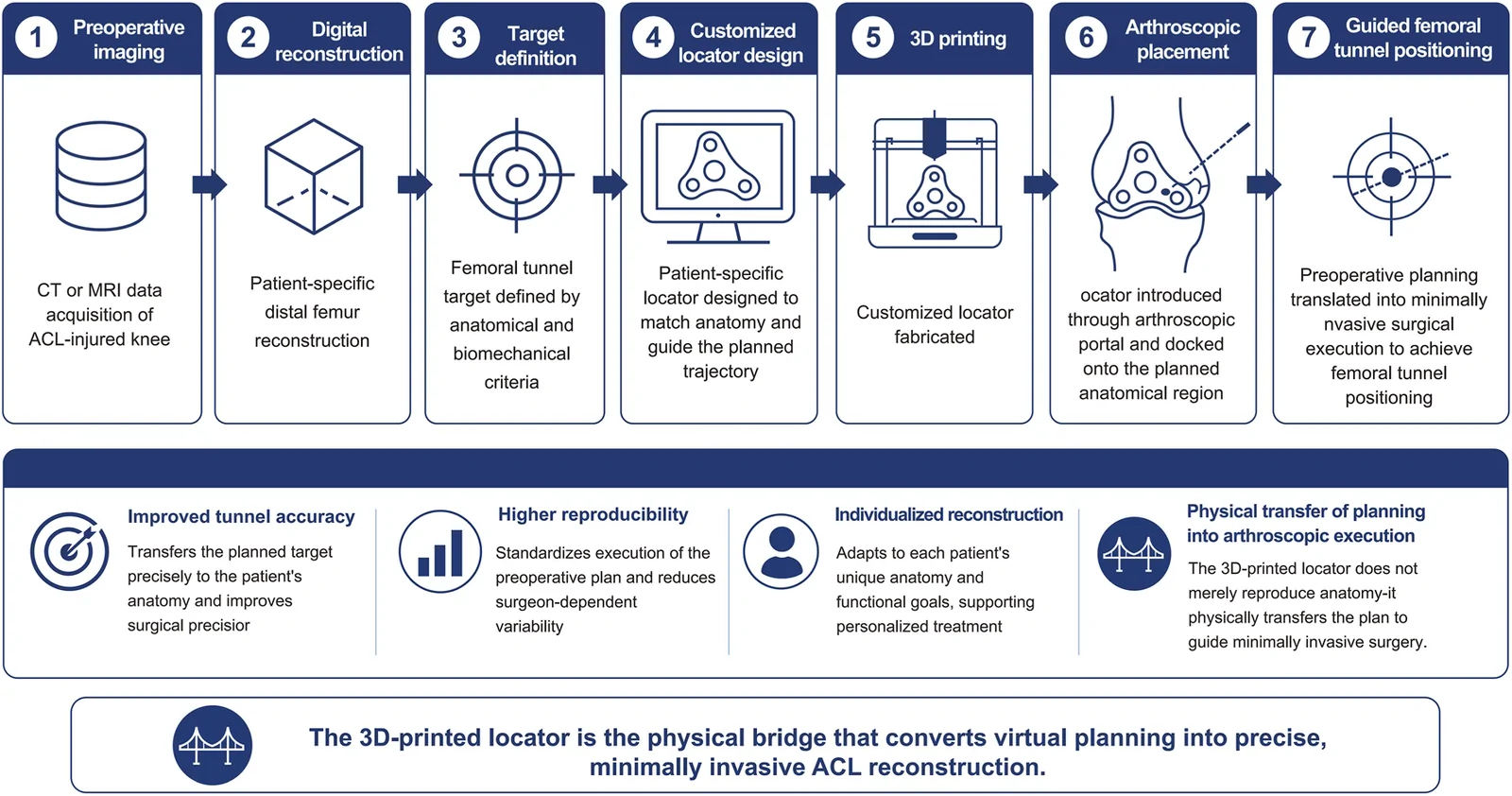
Translating preoperative digital planning into arthroscopic execution using a 3D-printed ACL locator. In patient-specific ACL reconstruction, preoperative CT or MRI data are used to reconstruct the distal femur and define the femoral tunnel target according to anatomical and biomechanical criteria. A customized 3D-printed arthroscopic locator is designed to match the lateral femoral condyle and guide the planned drilling trajectory. During arthroscopy, the locator is introduced through the portal, docked onto the planned anatomical region, and used to assist femoral tunnel positioning. This example illustrates the procedural value of digital manufacturing: the printed device does not simply reproduce anatomy, but physically transfers preoperative planning into minimally invasive surgical execution, potentially improving tunnel accuracy, reproducibility, and individualized reconstruction.

At the same time, clinical evidence for patient-specific ACL locators remains limited, and most available studies still focus on feasibility, technical accuracy, or early procedural outcomes rather than long-term comparative clinical results.

This example is conceptually important for four reasons. First, it demonstrates that patient-specific printing is not restricted to open reconstructive procedures. Second, it shows that temporary printed tools may have significant procedural value even when they are not implanted. Third, it highlights a new design problem: arthroscopic locators must be compact enough to enter a joint, stable enough to maintain registration, and safe enough to avoid cartilage injury. Fourth, it suggests that digital manufacturing may serve as a low-cost bridge between conventional surgical guides and more complex navigation or robotic systems.

3D-printed ACL locators should not be viewed simply as another application of surgical guides. They reveal a broader direction for the field: patient-specific devices can become instruments for procedural standardization. In settings where surgical outcome depends on precise localization, digital manufacturing may reduce variability, support training, and improve the reproducibility of complex maneuvers (Schweiger et al., 2021). To move beyond technical feasibility, a prospective pilot comparative study should directly compare patient-specific 3D-printed arthroscopic locators with conventional anatomical landmark-based or standard-guide techniques for ACL femoral tunnel placement (Wang et al., 2024). The primary endpoint should be tunnel placement accuracy, quantified on postoperative three-dimensional CT by the deviation between the planned and achieved tunnel center and drilling trajectory (Chen et al., 2024). Secondary procedural endpoints should include operative time, time required for femoral tunnel localization, number of positioning or redocking attempts, intraoperative device-related difficulties, and procedure-related complications. Short-to mid-term exploratory outcomes could include instrumented anterior knee laxity, pivot-shift testing, International Knee Documentation Committee scores, Lysholm scores, Tegner activity scores, and graft-related complications assessed at approximately 3, 6, and 12 months (Al-Naseem et al., 2025). Postoperative imaging should be evaluated by assessors blinded to the surgical technique, and both groups should follow standardized surgical and rehabilitation protocols. Because a pilot study may not be sufficiently powered to establish differences in functional outcomes, its principal purpose should be to assess feasibility, estimate effect sizes and outcome variability, and inform the design of a larger prospective comparative trial.

Although ACL reconstruction is used here as the representative example, this paradigm may also be relevant to other arthroscopic procedures, such as shoulder or hip arthroscopy, where accurate localization of bony landmarks, anchor placement regions, or resection targets is required in a constrained operative field (Murphy and Wong, 2021). Similar principles may also apply to selected non-orthopedic minimally invasive procedures when preoperative imaging identifies a target that is difficult to localize directly during laparoscopy, endoscopy, or thoracoscopy (Tang et al., 2022). However, translation to these settings would require procedure-specific redesign according to the access route, working space, tissue mobility, docking surface, and safety constraints.

### 3.3 The next-generation will be hybrid rather than purely printed

Static printed arthroscopic locators have clear advantages, but they also have limitations. An arthroscopic guide designed from preoperative imaging assumes that intraoperative anatomy, exposure, and tissue conditions correspond sufficiently to the model. Arthroscopy introduces additional challenges, including fluid distension, soft-tissue obstruction, variable visualization angles, and limited working space (LaPrade et al., 2021). An arthroscopic locator may fit well in a model but be difficult to deploy in a real joint. These issues suggest that future systems may need to combine printed guidance with dynamic intraoperative technologies.

Hybrid guidance systems are therefore a promising direction. A printed arthroscopic locator can provide tactile anatomical registration, while navigation, augmented reality, optical tracking, or arthroscopic video-based analysis can provide real-time verification (Hu et al., 2022). In ACL reconstruction, such a system might use a patient-specific locator to define the target region and digital navigation to confirm trajectory (Zhang et al., 2025). This would preserve the simplicity of physical locators while reducing the risk of static mismatch.

However, such hybrid systems also face practical barriers in routine clinical use. Reliable registration must be maintained among the printed locator, patient anatomy, and digital navigation space, while calibration error, tracking drift, line-of-sight interruption, fluid interference, and soft-tissue obstruction may reduce accuracy. In addition, setup time, equipment cost, sterilization of tracked components, operating-room space, and staff training requirements may limit adoption unless the added digital layer provides clear procedural value.

We believe that the most clinically useful systems will not necessarily be the most technologically complex. Rather, they will be those that solve specific procedural problems while remaining compatible with routine surgical workflow. For knee arthroscopy, this means compact design, intuitive docking, minimal portal modification, rapid sterilization, and reliable intraoperative use (LaPrade et al., 2021). The ultimate goal is not to make the procedure look more digital, but to make it more accurate, reproducible, and patient-specific.

## 4 Therapeutic constructs should be judged by biological execution, not structural complexity

### 4.1 From passive scaffolds to therapeutic systems

Therapeutic constructs expand the role of 3D printing beyond mechanical reconstruction (Capotorto et al., 2025). In this context, a printed product may support tissue regeneration, deliver drugs, regulate inflammation, guide cell organization, or interact with a pathological microenvironment. Examples include porous bone scaffolds, osteochondral constructs, cartilage scaffolds, drug-releasing implants, antibacterial devices, cell-laden bioprinted tissues, and smart responsive materials (Wang et al., 2021; Zhao et al., 2025). These applications are particularly relevant to regenerative medicine because they allow geometry, architecture, material composition, and biological signals to be spatially organized.

However, the ability to print complex structures should not be confused with therapeutic success. A scaffold with sophisticated architecture may still fail if it does not support cell survival, matrix deposition, vascularization, mechanical maturation, or host integration (Osmond et al., 2020). A drug-loaded construct may be ineffective if the release profile is poorly controlled or if printing conditions damage the therapeutic agent (Lee et al., 2022). A bioprinted tissue may appear promising *in vitro* but fail to mature into a clinically durable structure (Ke et al., 2022). Therefore, therapeutic constructs should be evaluated according to biological execution rather than structural complexity alone.

We propose that a therapeutic construct should be designed backward from the clinical problem. An infected bone defect may require mechanical support, local antibacterial activity, osteoconductivity, and control of inflammatory remodeling (Li X. et al., 2026). A cartilage defect may require shape conformity, chondrogenic support, resistance to mechanical collapse, and integration with surrounding cartilage (Xuan et al., 2020). A minimally invasive orthopedic repair construct may require deliverability through a small portal, rapid stabilization, and compatibility with arthroscopic handling (Liu et al., 2025). This problem-first approach is essential if digital manufacturing is to produce clinically useful therapies rather than technically impressive prototypes.

### 4.2 Bioprinting must become more predictive and controllable

Bioprinting adds another layer of complexity by incorporating living cells into digitally manufactured constructs. The promise is clear: cell-laden bioinks can be patterned into patient-specific shapes, gradients, or tissue-like architectures (Van Belleghem et al., 2020). In cartilage and osteochondral repair, bioprinting may allow different cell types, matrix components, and mechanical domains to be arranged according to the biological requirements of the defect (Liang et al., 2022). This could eventually enable constructs that reproduce not only external geometry but also internal tissue organization.

Yet the translational distance remains substantial. Cell viability, bioink printability, mechanical stability, tissue maturation, vascularization, sterility, and reproducibility all remain difficult to control. Living constructs are inherently more variable than inert devices, and their quality cannot be defined by geometry alone. For this reason, bioprinting requires stronger integration with predictive modeling and non-destructive assessment. Before implantation, computational tools could estimate nutrient diffusion, mechanical stress distribution, and degradation behavior. After fabrication, imaging or sensor-based monitoring could evaluate construct quality. Over time, machine learning could connect printing parameters with biological outcomes.

In practical terms, this predictability may be improved by defining the intended biological endpoint before printing, such as matrix deposition, vascularization, tissue-specific differentiation, or controlled degradation (Son et al., 2025). The printing process should then be standardized around key quality attributes, including bioink consistency, cell viability, construct geometry, and early mechanical stability (Van Belleghem et al., 2020). After fabrication, longitudinal biological, mechanical, and imaging-based assessment can help determine whether the construct is progressing toward its intended therapeutic function (Liang et al., 2022).

Bioprinting will become clinically meaningful only when it becomes more predictable. The key challenge is not simply to print cells in a desired shape, but to guide those cells toward a stable and functional tissue state. This requires a shift from printing as placement to printing as biological programming.

## 5 Artificial intelligence and digital twins should serve clinical endpoints

Artificial intelligence is increasingly discussed as a natural partner for 3D printing (Zhang and Zhang, 2025). It can assist with image segmentation, anatomical landmark detection, implant design, scaffold topology optimization, structure–property prediction, process monitoring, and postoperative outcome analysis (Pugliese and Regondi, 2022). Recent deep-learning studies in multimodal medical imaging have demonstrated automated image registration and segmentation, as well as cross-attention-based image fusion, further supporting AI-enabled extraction and integration of patient-specific imaging information (Reddy and Nelakuditi, 2025; Sreekala et al., 2026). These tools are particularly relevant because patient-specific manufacturing increases design complexity. If every device is individualized, manual planning becomes time-consuming and potentially inconsistent. AI may help reduce this burden.

However, AI should not be added to digital manufacturing merely to increase technological sophistication. Its value should be judged by whether it improves clinically relevant decisions (Chen X. et al., 2025). For example, can AI reduce segmentation time without compromising anatomical accuracy? Can it identify stable docking regions for arthroscopic locators? Can it predict whether a scaffold will have sufficient mechanical strength and pore connectivity? Can it detect manufacturing defects before clinical use? Can it link postoperative outcomes to design parameters in a way that improves future devices? These are the kinds of questions that make AI clinically meaningful.

Digital twins offer a related but distinct opportunity. A digital twin could simulate how a patient-specific implant, surgical guide, or therapeutic construct behaves under patient-specific conditions (Cremese et al., 2025). In orthopedics, such models could estimate load distribution, tunnel trajectory, graft orientation, implant stress, scaffold degradation, or tissue ingrowth. They may also be updated using postoperative imaging, gait analysis, or patient-reported outcomes (Maksymenko et al., 2023). In this way, digital twins could help connect design, intervention, and follow-up.

Nevertheless, both AI and digital twins require caution. Clinical device design demands traceability, explainability, validation, and risk control (Park et al., 2021). This is also consistent with the modern regulatory concept of Software as a Medical Device (SaMD), which recognizes that software intended for medical purposes may itself require regulatory oversight even when it is not part of a physical device (Spanou, 2024). In patient-specific manufacturing, CAD software, AI-based segmentation tools, surgical planning platforms, and simulation systems may directly influence implant geometry, guide trajectory, target definition, or therapeutic construct design. These digital components may therefore face regulatory scrutiny regarding intended use, risk classification, validation, cybersecurity, traceability, and post-deployment performance (MDR and Regulation EU, 2017/746, 2017). A black-box model that produces a device geometry without documenting assumptions or uncertainty is unlikely to gain surgical or regulatory trust. Similarly, a digital twin that is not validated against real outcomes may create false confidence. AI and digital twins should therefore be developed within a quality-by-design framework (Sun et al., 2022). Their role should be to support safer, more reproducible, and more evidence-based intervention—not to replace clinical judgment. Figure 2 summarizes this proposed digital manufacturing workflow. In this framework, patient imaging, segmentation, target definition, CAD design, AI-assisted optimization, 3D printing, post-processing, clinical application, and outcome assessment are linked through an iterative loop. This model emphasizes that the printed product is not the final endpoint. Rather, it is one component of a learning system in which clinical outcomes inform future design.

**FIGURE 2:**
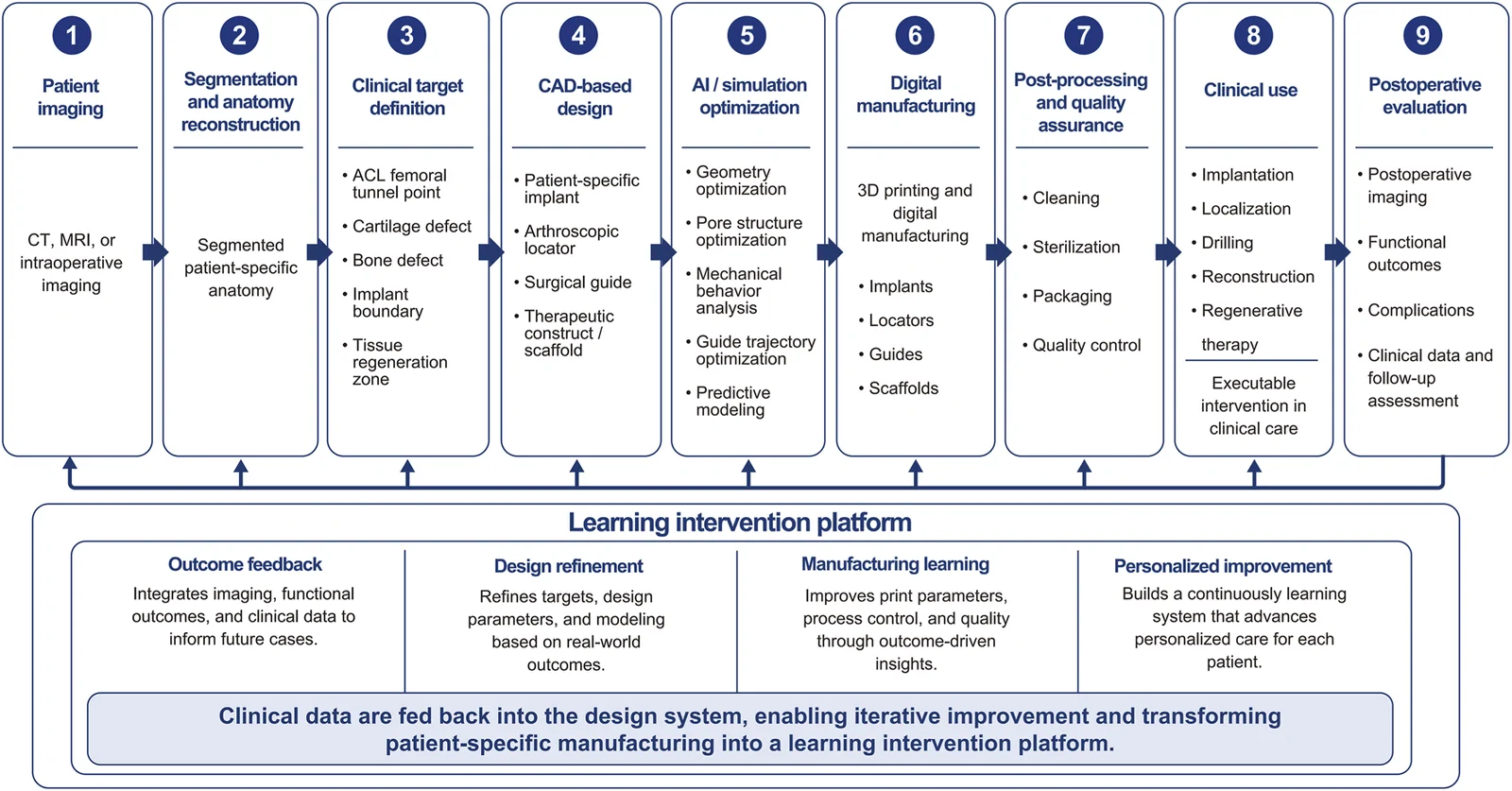
Digital-to-clinical workflow for patient-specific manufacturing and executable intervention. The figure reframes 3D printing as part of an integrated digital-to-clinical system rather than an isolated fabrication step. Patient imaging is converted into segmented anatomy, clinical target definition, CAD-based design, and AI- or simulation-assisted optimization. Digital designs are then manufactured as patient-specific implants, surgical guides, arthroscopic locators, or therapeutic constructs, followed by post-processing, sterilization, and quality control. During clinical use, these products support implantation, localization, drilling, reconstruction, or regenerative therapy. Postoperative imaging, functional outcomes, complications, and clinical data are fed back into the design system, enabling iterative improvement and transforming patient-specific manufacturing into a learning intervention platform.

However, such feedback loops also face practical barriers. Across hospitals, imaging protocols, electronic health records, CAD files, manufacturing records, and outcome measures are often not fully standardized, which makes multicenter data integration difficult. Patient-specific imaging, anatomical models, and implant-design files may also raise privacy concerns because they can contain highly individualized anatomical information. Therefore, learning manufacturing systems will require interoperable data standards, secure data governance, and privacy-preserving approaches before outcome-informed design can be widely implemented.

### 5.1 Validation, explainability, and outcome-informed updating

Testing of AI tools should begin with a clearly defined intended use, target population, input data, expected output, user role, and clinical consequence of an incorrect result. Development, tuning, and test datasets should be separated at the patient level, with final performance evaluated on a locked test set that has not influenced model development. External, temporal, and preferably multicenter validation should then assess generalizability across imaging devices, acquisition protocols, institutions, operators, anatomical variants, and clinically relevant patient subgroups (Tejani et al., 2024). The selected performance metrics should reflect the clinical task. Segmentation models should be evaluated using overlap and surface-distance measures together with errors at critical anatomical landmarks. Landmark-detection and design-support tools should be assessed by deviations in entry points, trajectories, docking surfaces, wall thickness, or other critical geometric features relative to clinician-approved references. Predictive models should report discrimination, calibration, clinically relevant error rates, and performance across prespecified subgroups. Prospective human–AI evaluation should additionally determine whether the tool reduces planning time or variability without increasing clinically important errors (Vasey et al., 2022).

Digital twins require a related but distinct credibility framework. Model verification should confirm that the equations, software implementation, mesh resolution, and numerical procedures perform as intended. Calibration should estimate patient-specific parameters using appropriate anatomical, mechanical, or physiological data, while validation should compare simulated outputs with independent benchtop, phantom, cadaveric, intraoperative, or postoperative measurements. Sensitivity analysis and uncertainty quantification should identify which assumptions and input variables most strongly influence clinically relevant predictions. The required validation rigor should be proportional to the role of the digital twin in decision-making and the potential harm caused by an incorrect prediction. A model used for exploratory visualization may require less evidence than one used to determine implant geometry, drilling trajectory, mechanical loading, or therapeutic delivery.

Explainability should be designed for the intended user and clinical context rather than reduced to a single visualization. Surgeons and engineers should be able to review the provenance and quality of the input data, the anatomical landmarks or design constraints that influenced the output, model confidence or uncertainty, the conditions under which the model was validated, and the circumstances in which human review or override is required (Health, 2024). Relevant limitations should also be stated explicitly. AI performance may deteriorate because of domain shift, imaging artifacts, incomplete anatomy, rare anatomical variants, imbalanced training data, changes in clinical practice, or propagation of upstream segmentation errors. Digital twins may be limited by simplified boundary conditions, uncertain tissue properties, incomplete representation of biological adaptation, absence of real-time physiological data, and accumulated uncertainty across model components. Out-of-distribution inputs and low-confidence outputs should therefore trigger review or deferral rather than automatic clinical execution (FUTURE-AI, 2025).

A practical postoperative learning system could be established through a prospective registry linked to the hospital imaging, clinical, and manufacturing records (Zinchenko et al., 2022). Each case should receive a unique traceable identifier connecting the source imaging, segmentation and CAD versions, AI or digital-twin outputs, manufacturing and quality-control records, intraoperative use, and postoperative outcomes. Core data should include planned and achieved anatomical or procedural parameters, operative time, intraoperative modifications, device-related difficulties, complications, revisions, postoperative imaging, functional outcomes, and patient-reported outcome measures collected at predefined procedure-specific intervals. For ACL reconstruction, for example, these data could include tunnel-position deviation, knee stability, graft-related complications, and IKDC, Lysholm, and Tegner scores (Abed et al., 2024), collected at approximately 3, 6, and 12 months. Data definitions, imaging protocols, follow-up windows, missing-data procedures, privacy controls, and outcome adjudication should be standardized before collection.

New postoperative data should not be allowed to change a deployed model automatically. Instead, performance should be monitored for calibration drift, changes in error rates, subgroup disparities, and previously unrecognized failure modes (Health, 2025). Candidate updates should be developed in a separate environment, evaluated first in silent or shadow mode, and tested using locked temporal and external datasets before clinical deployment. The updated model should receive a new version number, retain an audit trail and rollback mechanism, and undergo documented risk assessment and revalidation. Where applicable, anticipated modifications, update methods, acceptance criteria, and impact assessments may be specified through a predetermined change-control plan. This controlled approach allows postoperative evidence to support iterative improvement without allowing continuous learning to compromise traceability, safety, or the validity of the previously approved workflow.

## 6 Discussion

### 6.1 Translational success requires workflow validation

Patient-specific digital manufacturing is now technically mature enough to generate customized implants, surgical guides, arthroscopic locators, scaffolds, and therapeutic constructs. However, technical feasibility should not be confused with translational readiness. The major challenge is no longer whether a device can be printed in a laboratory. The challenge is whether the entire workflow can be standardized, validated, reproduced, regulated, and integrated into clinical care.

Quality control is central to this transition. Patient-specific devices challenge conventional manufacturing models because each product may be geometrically unique. Even when the same printer, material, and process are used, device performance can be affected by segmentation accuracy, design choices, build orientation, support removal, post-processing, sterilization, and final inspection. For implants, mechanical testing, fatigue behavior, surface characteristics, biocompatibility, and dimensional accuracy are critical (Bandyopadhyay et al., 2023). For surgical guides and arthroscopic locators, fit, stability, sterilization compatibility, and workflow usability are essential (Food and Drug Administration Staff, 2020). For therapeutic constructs, biological activity, release kinetics, degradation, and cell behavior must also be assessed.

Regulation and point-of-care manufacturing add further complexity. Hospitals may increasingly produce anatomical models, guides, and selected devices close to the point of care. This could shorten turnaround time and improve surgeon-engineer communication, but it also raises questions of responsibility, documentation, material qualification, printer validation, and adverse event traceability. Recent regulatory discussions have begun to address these issues. For example, the FDA has issued a discussion paper on 3D printing medical devices at the point of care, and the EU Medical Device Coordination Group has provided guidance on the health institution exemption under Article 5(5) of the MDR/IVDR (Health, 2021). These documents indicate that hospital-based manufacturing should still be considered within a risk-based framework, with attention to quality systems, device classification, intended use, and accountability. A printed anatomical model for planning does not have the same risk profile as an implantable drug-releasing scaffold. Therefore, regulatory pathways must be risk-based and product-specific.

Clinical evidence also remains uneven. Many studies report feasibility, accuracy, or early outcomes, but broader adoption will require prospective evaluation, standardized endpoints, and cost-effectiveness analysis. The economic case for patient-specific manufacturing is likely to be context-dependent. Customized implants, guides, or locators may reduce intraoperative adjustment, shorten operative time, improve procedural accuracy, or potentially lower revision-related costs. However, these potential savings must be weighed against the upfront costs of image processing, segmentation, design labor, software licensing, printer maintenance, materials, sterilization, quality control, and regulatory documentation (Ballard et al., 2020). Patient-specific manufacturing may be more economically justifiable in complex cases where conventional devices are poorly suited, or when improved accuracy can prevent costly complications or revisions. In routine procedures where mass-produced devices already perform reliably, its economic advantage remains uncertain and should be tested through procedure-specific cost-effectiveness analyses. For ACL locators, for example, it will be important to determine whether improved tunnel positioning translates into better rotational stability, functional scores, lower revision rates, or improved learning curves (Gong and Yin, 2025). For therapeutic constructs, the key question is whether printed biological systems produce durable tissue regeneration under clinically realistic conditions (Son et al., 2025). Without such evidence, 3D printing risks remaining a promising but fragmented innovation.

### 6.2 Standardized protocols and product-specific release criteria

Standardization should be applied to the digital manufacturing workflow rather than to patient-specific geometry. A minimum protocol should prospectively document each stage from image acquisition to final product release. For imaging acquisition, studies should report the imaging modality, scanner and acquisition protocol, patient positioning, field of view, slice thickness or voxel spacing, reconstruction kernel or magnetic resonance imaging sequence, and predefined criteria for acceptable motion, metal, or partial-volume artifacts. For segmentation and digital design, the software and version, automated or manual segmentation method, threshold or model settings, manual corrections, operator qualifications, mesh repair and smoothing procedures, anatomical landmarks, docking surfaces, planned trajectories, and independent clinical approval of the final design should be recorded. Digital files should be version-controlled so that the approved patient anatomy, computer-aided design model, printing file, and final manufactured product remain traceable (Food and Drug Administration Staff, 2021).

Build preparation and printing records should include the printer and slicing-software versions, material type and batch, build orientation, support strategy, nesting position, layer thickness, and process-specific settings. Depending on the manufacturing modality, these settings may include nozzle diameter, extrusion temperature, infill and printing speed for material extrusion; or laser power, scan speed, hatch spacing, chamber atmosphere, and preheating conditions for powder-bed fusion. Post-processing records should specify support removal, cleaning, washing, curing, thermal treatment, machining, surface finishing, and inspection steps. Sterilization should be selected according to material compatibility and intended clinical use, with documentation of the sterilization modality, cycle parameters, load configuration, packaging, validated sterility controls, and any dimensional, mechanical, chemical, or biological changes caused by the sterilization process.

Validation of unique patient-specific products should combine process-level validation with per-product verification (Wg, 2023a). Process validation should qualify the equipment, material, validated parameter window, representative or worst-case geometries, post-processing procedures, and sterilization cycle. When destructive testing is required, standardized test coupons or companion specimens manufactured in the same build may be used to assess material and mechanical properties. Each patient-specific product should then undergo non-destructive verification against the clinically approved digital model. Recommended quality-control metrics include global and local surface deviation, critical-feature dimensional error, entry-point and trajectory deviation, docking gap or contact area, internal defects or porosity, surface roughness, mechanical integrity, residual material or contamination, and sterilization and biological-safety indicators where applicable.

Acceptance limits should be predefined according to the intended use, product risk, manufacturing capability, and clinical consequence of deviation rather than applying one universal tolerance to all printed products. For an arthroscopic locator, for example, the critical-to-quality attributes would include docking-surface conformity, guide-hole diameter, planned-versus-manufactured entry-point deviation, trajectory angular deviation, structural integrity, smoothness of tissue-contacting surfaces, and dimensional stability after sterilization. These metrics should be incorporated into a documented release checklist. A product should be released for clinical use only when all critical attributes remain within the predefined limits; an out-of-specification result should trigger documented investigation, rework followed by repeat verification, or product rejection. This approach allows geometrically unique devices to be manufactured within a validated process while ensuring that their patient-specific clinical features are independently verified before use. The minimum reporting items, quality-control metrics, and release applications for patient-specific digital manufacturing are summarized in Supplementary Table S2.

### 6.3 Material, software, and workflow risk management

Material and product testing should be embedded within a formal risk-management process rather than treated as an independent preclinical checklist. The process should begin by defining the intended use, patient population, anatomical site, duration of contact, load-bearing requirements, mode of clinical deployment, and foreseeable misuse. Hazards should then be identified across the complete workflow, including imaging artifacts, segmentation or landmark errors, assignment of the wrong patient dataset, inappropriate material selection, inadequate wall thickness, build-orientation anisotropy, incomplete fusion or internal defects, residual powder or resin, dimensional changes during post-processing or sterilization, mechanical failure, wear or corrosion products, degradation-related toxicity, infection, and inaccurate intraoperative positioning. A structured method, such as failure mode and effects analysis, may be used to estimate the severity and probability of harm, define risk-control measures, verify the effectiveness of those controls, and evaluate whether the remaining residual risk is acceptable in relation to the expected clinical benefit (Jiang et al., 2024).

The regulatory pathway should be determined by intended use and product risk rather than by the fact that the product is patient-specific. An anatomical model used only for visualization, a temporary arthroscopic locator, a permanent load-bearing implant, a drug-releasing scaffold, and a cell-laden construct may require different levels of design control, biological evaluation, mechanical validation, clinical evidence, and regulatory oversight (Wg, 2023b). Patient specificity does not remove the need to establish material specifications, manufacturing controls, clinical performance, labeling, traceability, and post-deployment surveillance. Products incorporating drugs, viable cells, or biologically active components may also require evaluation as combination products or under additional medicinal-product or biologic frameworks, depending on the jurisdiction and principal mode of action. Selected regulatory pathways relevant to point-of-care manufacturing are summarized in Table 1. Because these pathways are jurisdiction-specific and continue to evolve, health institutions should confirm the applicable requirements with the relevant competent authority before clinical implementation.

**TABLE 1:** Selected regulatory pathways relevant to point-of-care manufacturing of patient-specific medical products.

Jurisdiction or framework	Potential regulatory pathway	Principal requirements and restrictions	Practical implication for point-of-care manufacturing
United States—FDA	Existing device pathway according to classification and intended use, including 510(k), de novo, or premarket approval; the custom device exemption applies only in narrowly defined circumstances	Evidence requirements depend on device risk and intended use. Additive manufacturing requires control of design, material, build process, post-processing, dimensional accuracy, mechanical performance, cleaning, and sterilization. The FDA’s 2021 point-of-care document is a discussion paper rather than final guidance	A hospital should not assume that local manufacture creates a regulatory exemption. Responsibility should be defined according to who controls the device design, manufacturing specifications, validation, and release
European Union—MDR article 5(5)	Health-institution exemption for qualifying in-house devices; alternatively, the conventional CE-marked or custom-made-device pathway may apply	A qualifying in-house device must be manufactured and used only within an EU health institution, must not be transferred to another legal entity, must meet the relevant annex I general safety and performance requirements, and requires an appropriate quality-management system, documented justification, technical documentation, a public declaration, and review of clinical experience. Custom-made devices are outside article 5(5) and follow separate MDR requirements	The institution must determine early whether the product is an in-house device, a custom-made device, or a conventionally regulated device. Manufacture for transfer to an independent hospital or legal entity generally prevents reliance on article 5(5)
Great Britain—UK MDR 2002 and MHRA guidance	Health institution exemption, custom-made-device route, or full conformity-assessment pathway, depending on intended use and manufacturing circumstances	The institution should formally determine whether the health institution exemption applies or whether the product is custom-made or requires full regulatory assessment. Current MHRA guidance emphasizes governance, safety, documentation, traceability, and use for the health institution’s own patients. Custom-made devices remain subject to applicable United Kingdom MDR requirements, documentation, statements, and registration obligations	“Made in a hospital” is not by itself sufficient to establish exemption. The regulatory route, responsible manufacturer, clinical use, and transfer arrangements must be documented before manufacture
Australia—TGA personalised medical-device framework	Adaptable, patient-matched, custom-made, or conventional medical-device pathway	Most personalised devices that were previously regarded as custom-made are now classified as patient-matched devices. Transitional exemption from inclusion in the australian register of therapeutic goods applies to eligible patient-matched devices until 1 July 2029. Review of point-of-care regulation remains ongoing, and the medical device production system concept is not yet fully activated	Hospitals should classify the product under the current personalised-device framework and verify whether a transitional exemption applies. Future PoC-specific changes may alter responsibilities for end-to-end manufacturing systems
International technical standards	Supporting quality and validation framework rather than an independent market-authorisation pathway	Relevant standards may include ISO 13485 for quality management, ISO 14971 for risk management, ISO/ASTM 52920 for additive-manufacturing process qualification, ISO 10993-1 for biological evaluation, and IEC 62304 when medical-device software forms part of the workflow	Standards can support process validation, risk control, software governance, biological evaluation, and audit readiness, but compliance with standards does not by itself replace jurisdiction-specific regulatory authorization

Products incorporating drugs, viable cells, gene-modified components, or a primary pharmacological, immunological, or metabolic mode of action may fall under combination-product, medicinal-product, biologic, tissue-engineered-product, or advanced-therapy frameworks rather than a medical-device-only pathway.

Software used for image segmentation, anatomical reconstruction, target definition, CAD design, trajectory planning, simulation, or AI-assisted optimization should be included in the risk-management and validation boundary whenever its output can influence the geometry or clinical function of the final product (Sun et al., 2023). Required controls should include software-intended-use definition, version locking, access control, verification of data transfer and file conversion, validation using representative and challenging datasets, documentation of algorithm or threshold settings, independent clinical review of patient-specific outputs, cybersecurity and data-integrity controls where applicable, and predefined procedures for software updates. An update to the segmentation algorithm, CAD software, printer-control software, or file-conversion process should trigger a documented assessment of whether partial or complete revalidation is required (FDA Staff, 2020).

End-to-end traceability should connect the source imaging dataset, patient identifier, segmentation file, approved anatomical model, design requirements, CAD version, risk controls, manufacturing file, software versions, material and batch, printer and build record, post-processing and sterilization records, inspection results, and final clinical release decision. A traceability matrix can link each clinical and design requirement to the associated hazard, risk-control measure, verification method, acceptance criterion, and release record. Overall workflow validation should then demonstrate that the integrated process—not only the printer—reliably converts an approved patient dataset into a product that meets its predefined critical-to-quality attributes. Validation should use representative and worst-case datasets or geometries, assess operator-dependent steps, challenge data-transfer interfaces, and confirm that failures can be detected before clinical release. The proposed material-specific tests, principal risks, and regulatory and validation considerations are summarized in Supplementary Table S3.

### 6.4 Economic and implementation feasibility

The economic value of patient-specific digital manufacturing should be assessed at both the individual-procedure and institutional-program levels. Annual fixed costs may include printer depreciation, software licenses, facility preparation, equipment maintenance and calibration, process validation, quality-management infrastructure, and personnel training. Variable costs should include image processing, segmentation and design labor, clinical review, printing materials, machine time, failed or repeated builds, post-processing, sterilization, packaging, inspection, documentation, and product release. Potential benefits may arise from reduced operating-room time, fewer positioning attempts, lower fluoroscopy or consumable use, avoidance of intraoperative device modification, shorter hospitalization, and, if demonstrated, fewer complications or revisions. A transparent local analysis can calculate per-case net value as the monetary value of these avoided resources minus the variable manufacturing cost, while annual net value should additionally subtract the fixed cost of maintaining the service. The annual break-even volume can then be estimated by dividing annual fixed costs by the expected net saving per case (Serrano et al., 2020).

As an illustrative benchmark, a previous literature-based analysis of orthopedic and maxillofacial applications reported that 3D-printed surgical guides were associated with an average reduction of approximately 23 min in operating-room time. Using an assumed operating-room cost of USD 62 per minute, the authors estimated a saving of approximately USD 1,488 per case and a break-even volume of approximately 63 printed models or guides per year (Ballard et al., 2020). These estimates are context-specific, based on historical United States cost assumptions, and should not be directly transferred to ACL arthroscopic locators or other patient-specific products. For the proposed ACL locator, a pilot comparative study should prospectively record staff time, design and manufacturing costs, operating-room time, consumable use, production failures, complications, revisions, and functional outcomes. This would allow calculation of local cost per successfully released locator, net cost or saving per case, budget impact at different annual case volumes, and, when adequately powered, incremental cost per clinically meaningful improvement or quality-adjusted life-year.

Implementation feasibility may nevertheless limit economic value even when the printed product itself is inexpensive. Point-of-care manufacturing requires trained personnel for segmentation, design, engineering review, printing, post-processing, sterilization, and quality assurance, together with clearly assigned clinical and manufacturing responsibilities. Additional barriers include inconsistent case volume, competing requests for printer capacity, long or unpredictable turnaround times, printer downtime, failed builds, limited availability of validated materials, post-processing and sterilization bottlenecks, physical-space and ventilation requirements, software interoperability, secure transfer of patient data, document control, regulatory liability, reimbursement uncertainty, and the need to coordinate surgeons, engineers, radiologists, sterile-processing personnel, and quality-management teams. These additional steps may offset operating-room savings when the workflow is poorly integrated or when the product is unavailable by the scheduled procedure (Bastawrous et al., 2022).

A practical implementation strategy should therefore begin with a centralized, multidisciplinary service and a limited set of clearly defined clinical indications (Hellman et al., 2023). Standardized electronic ordering, case-selection criteria, responsibility matrices, backup manufacturing or outsourcing pathways, and target turnaround times should be established before routine use. Operational performance should be monitored using metrics such as order-to-release time, professional labor hours per case, first-pass production yield, reprint rate, printer utilization, on-time delivery, sterilization or quality-control delays, deviations from approved protocols, and total cost per clinically released product. Adoption should be expanded only when predefined clinical, economic, and operational targets are met. This staged approach is likely to be more sustainable than attempting to introduce patient-specific manufacturing simultaneously across all procedures and product categories.

### 6.5 Future progress depends on redefining what counts as success

Our central argument is that patient-specific digital manufacturing should stop asking only whether customized devices can be printed and start asking whether patient-specific interventions can be executed and improved. This shift has practical consequences.

First, it changes what should be measured. Studies should evaluate not only anatomical fit, printing accuracy, or material properties, but also intraoperative usability, procedural reproducibility, biological performance, patient outcomes, workflow efficiency, and cost-effectiveness. Second, it changes what should be optimized. The goal should not be to maximize geometric complexity or material novelty, but to optimize the relationship between patient data, device design, clinical action, and outcome (Bolshakov et al., 2020). Third, it changes what should count as translational readiness. A device should be judged not only by laboratory performance, but by whether it can be manufactured reproducibly, sterilized safely, used reliably, and evaluated longitudinally (Zhang et al., 2026).

Future progress will depend on three converging priorities. The first is clinically defined design: every printed implant, surgical guide, arthroscopic locator, or therapeutic construct should be developed in response to a specific surgical or therapeutic bottleneck. The second is intelligent workflow integration: AI, simulation, digital twins, and feedback loops should be used to improve decision-making and quality control. The third is translational discipline: patient-specific manufacturing must become compatible with regulatory standards, hospital workflows, outcome registries, and evidence-based adoption.

If these priorities are pursued together, 3D printing may evolve from a fabrication technology into a core infrastructure for precision surgery and regenerative healthcare. If they are pursued separately, the field may continue to produce impressive customized devices without fully transforming clinical care.

### 6.6 Limitations of the current evidence

Several limitations should be considered when interpreting the proposed framework. First, the clinical evidence supporting many patient-specific implants, surgical guides, arthroscopic locators, and therapeutic constructs remains dominated by feasibility studies, retrospective cohorts, small prospective series, technical reports, and short-term follow-up. Long-term evidence regarding implant survival, revision risk, functional durability, biological integration, and late complications remains limited for many applications. This is particularly relevant to patient-specific ACL locators, for which improved tunnel-positioning accuracy has not yet been consistently linked to superior long-term knee stability, graft survival, return to activity, or lower revision rates. Accordingly, the present Perspective should be interpreted as a translational framework and research agenda rather than evidence that all patient-specific products are already clinically superior to conventional approaches. Substantial heterogeneity exists in imaging acquisition, reconstruction, segmentation, anatomical landmark definition, design software, printing modality, material selection, post-processing, sterilization, and outcome assessment. Differences in CT slice thickness, MRI sequences, reconstruction algorithms, artifact burden, and image quality may propagate through segmentation and design and ultimately affect the fit or accuracy of the final product. Studies also use different definitions and measurement methods for tunnel position, trajectory deviation, docking accuracy, procedural time, mechanical performance, and functional outcomes. These methodological differences limit direct comparison, meta-analysis, and the transfer of reported accuracy thresholds across institutions. Generalizability may be restricted by variation in patient anatomy, disease severity, bone and soft-tissue quality, surgeon experience, institutional infrastructure, software platforms, manufacturing expertise, and regulatory requirements. Results obtained in high-volume centers with dedicated engineering and quality-management teams may not be reproducible in hospitals with lower case volumes, limited imaging quality, fewer trained personnel, or outsourced manufacturing workflows. Similarly, findings from one anatomical region, material, printer, or clinical procedure should not be assumed to apply directly to other products with different mechanical, biological, or procedural requirements. Evidence regarding cost-effectiveness, workflow reliability, and point-of-care implementation remains context-dependent. Reported reductions in operative time or resource use may be offset by segmentation, design, printing, post-processing, sterilization, documentation, validation, and failed-build costs. Economic findings generated within one health system may not be transferable to institutions with different labor costs, reimbursement structures, case volumes, regulatory responsibilities, or access to manufacturing infrastructure. AI-assisted design and digital-twin approaches remain limited by dataset representativeness, domain shift, incomplete physiological information, uncertain model assumptions, and insufficient prospective validation. Postoperative learning systems also depend on standardized longitudinal data, reliable follow-up, interoperable records, and controlled model updating, which are not yet routinely available across institutions. These limitations reduce the immediate applicability of fully adaptive manufacturing systems and reinforce the need for multicenter prospective studies, harmonized reporting standards, longer follow-up, external validation, and procedure-specific clinical and economic evaluation.

## 7 Conclusion

Patient-specific 3D printing and digital manufacturing have progressed from anatomical modeling and customized implants to surgical guides, arthroscopic locators, bioactive scaffolds, bioprinted constructs, and intelligent manufacturing workflows. Orthopedic applications, including 3D-printed arthroscopic locators for knee arthroscopic ACL reconstruction, illustrate how digital manufacturing can transfer preoperative planning into the operative field and support more individualized procedures. Therapeutic constructs further expand this paradigm by linking printed architecture with biological regulation and regenerative function.

The next stage of the field should be defined by a broader concept of success. A printed device should not be valued only because it is customized, complex, or technically feasible. It should be valued because it enables a clinically meaningful intervention that is accurate, reproducible, safe, scalable, and capable of improvement through feedback. By integrating patient imaging, advanced materials, AI-assisted design, digital twin modeling, manufacturing quality control, and postoperative outcomes, patient-specific digital manufacturing may become a foundation for learning, adaptive, and precision-oriented health systems.

## Data Availability

The original contributions presented in the study are included in the article/Supplementary Material, further inquiries can be directed to the corresponding author.
